# Factors influencing trust among colleagues in hospital settings: a systematic review

**DOI:** 10.1186/s12913-024-12159-6

**Published:** 2025-01-03

**Authors:** Andreea Isabela Varga, Ivan Spehar, Frode Veggeland, Helge Skirbekk

**Affiliations:** 1 Department of Health Management and Health Economics, Institute of Health and Society, Medical Faculty, University of Oslo (UiO), Oslo, Norway; 2 Department of Organisation, Leadership and Management, University of Inland Norway, Lillehammer, Norway; 3 Norwegian Institute of Bioeconomy Research (NIBIO), Oslo, Norway; 4 Department of Nursing and Health Promotion, Faculty of Health Sciences, Oslo Metropolitan University, Oslo, Norway

**Keywords:** Trust, Trust relations, Colleagues, Peers, Co-workers, Healthcare professionals, Hospital, Systematic review

## Abstract

**Background:**

Many studies show positive results of collegial trust in the workplace, e.g. performance, innovation and collaboration. However, no systematic review on collegial trust in hospital settings exists. This study aimed to provide the missing overview of factors that positively and negatively influence this trust relationship between healthcare providers.

**Methods:**

Ten information sources (Web of Science, Embase, MEDLINE, APA PsycInfo, CINAHL, Scopus, EconLit, Taylor & Francis Online, SAGE Journals and Springer Link) were searched from database inception up until October 21st, 2022. Empirical studies included were written in English, undertaken in a hospital or similar setting, and addressed collegial trust relationships between healthcare professionals, without date restrictions. Studies were excluded if they only explored trust between healthcare professionals on different hierarchical levels. Theoretical studies, systematic reviews, conceptually unclear papers and anecdotal case studies were also excluded. Records were independently screened for eligibility by at least two researchers. A narrative synthesis technique was adopted to explore and discuss the influencing factors of trust between colleagues identified across both quantitative and qualitative studies. This method was chosen given the inclusion of studies with different research designs and the unsuitability of the data for a meta-analysis or meta-ethnography. Risk of bias was assessed independently by at least two researchers using four critical appraisal tools.

**Results:**

Eight thousand two hundred sixty-eight studies were screened and 11 studies were included. Seven were qualitative and four were quantitative. Themes identified were professional competence, elements of communication, such as tacit knowledge sharing, and ethical conduct, such as honesty, confidentiality and accountability. Moreover, trust among colleagues was seen to thrive in work environments characterised by psychological safety. The results of the quality assessment show that most studies were of an acceptable quality, with some associated risk of bias. One of the limitations was represented by the lack of a definition for trust in some studies, and some inconsistency for those studies that did define trust.

**Conclusions:**

Professionalism, communication and ethics were seen as the most important factors enhancing trust. However, these concepts were defined differently in the studies.

**Trial registration:**

PROSPERO; CRD42023433021.

**Supplementary Information:**

The online version contains supplementary material available at 10.1186/s12913-024-12159-6.

## Background

Trust in healthcare professionals has long been established as important for the treatment and well-being of the patient [1, 2]. But it is also a phenomenon of great importance for healthcare workplaces. In a recent paper, we reviewed trust between managers and healthcare professionals in hospital settings [3]. In this paper, we aim to systematically review studies on collegial trust among healthcare professionals.

Trust is a social phenomenon studied widely. An often used definition for this concept is proposed by Mayer, Davis and Schrooman: “the willingness of a party to be vulnerable to the actions of another party based on the expectation that the other will perform a particular action important to the trustor, irrespective of the ability to monitor or control that other party”[4].

Taylor and colleagues’ systematic review [5] revealed that one of the seven factors associated with high performing hospitals consisted of a positive organisational culture. And one of the five characteristics of this factor is “respect and trust between colleagues at all levels in clinical and non-clinical services” [5].

In this systematic review we will study the trust relationships among employees working as colleagues; a topic that has been widely studied in various organisations such as the public sector [6–9], in higher education settings [10], and in healthcare settings [11–17].

The presence of such trust relationships can have several positive outcomes. In a UK survey study, interpersonal trust among local government employees “had a direct impact on organisational performance” [7]. In another large survey study on around 2500 employees working in the public sector from 18 Latin American countries showed that when trust among colleagues is high, their willingness for collaboration, information sharing and support for technological innovations increased, and their mission motivation improved [9]. Similarly, another study conducted on public sector employees in Kenya concluded that “coworker trust was positively related to knowledge sharing” [6].

Positive effects of trust among co-workers have also been explored within healthcare settings. A study from South Korea showed that trust was one of the factors positively influencing the intention and behaviour of sharing knowledge; as well as the innovation behaviour of employees (nurses, administrative staff and medical technicians) working in four university hospitals [18]. In Taiwan, a survey study on 400 registered nurses working in a teaching hospital showed that trust among co-workers had a significant and positive effect on organisational commitment [19]. Similarly, another study established that trust among nurses working in a private hospital in Istanbul was in a positive and significant relationship with what the authors called organisational citizenship behaviour, encompassing aspects such as conscientiousness, sportsmanship, civic virtue and altruism [16]. A very large survey of nurses (*N* = 9000) working in China revealed that the levels of trust between nurses and physicians is “significantly associated with higher job satisfaction scores” [20]. Similarly, in a study conducted on nurses (*N* = 155) working in comprehensive nursing care service units in Seoul, trust among colleagues alongside organizational intimacy and communication abilities were factors that positively influenced job satisfaction [21]. In a mixed-methods study on enrolled nurses (EN) and registered nurses (RN) working as a team in Singapore; the qualitative data showed that trust between RNs and ENs was an important interpersonal factor for good teamwork, and lack of trust had a negative impact on their work focus and efficiency, creating a stressful working environment [22].

While establishing and maintaining trust relationships among hospital employees working as colleagues have positive outcomes, the absence of them can have negative consequences. For example, a qualitative study compared organizational differences between patient care units with high and low levels of missed nursing care. They identified trust as one of the 10 themes contrasting the two units. Staff trusted one another in the nursing care units with the highest levels of nursing care [23]. In a survey study on psychological issues among registered nurses working in the United States, it was revealed that psychological safety, which is a scale measuring team trust and respect, “was inversely associated with being personally bullied and witnessing bullying” [24].

Given the positive consequences of trust and the negative consequences of mistrust relationships outlined above, both for healthcare delivery and for their well-being as hospital employees, this systematic literature review will identify key factors that might promote or impede trust among healthcare providers. Hospital managers can use these insights to either strengthen or establish trustful relationships within their teams.

A recent scoping review mapped the empirical and grey literature on how trust within healthcare teams is defined and measured, as well as what the antecedents and consequences of this relationship are [25]. The authors focused specifically on healthcare teams in a broader range of settings than we aim for, from primary care to tertiary care. However, there is to our knowledge no systematic literature review conducted that provides an overview of the causes or influencing factors of peer trust relationships in hospital settings.

This systematic literature review aims to explore the causes of such collegial trust relationships found in the published literature, and to answer the following research question:

*What are the causes or influencing factors of trust between healthcare professionals working as colleagues in a hospital or similar setting?*

The review is not focused on a particular set of factors as it aims to include aspects ranging from organizational factors to interpersonal and psychological attributes.

## Methods

This systematic literature review started out as a multifaceted project related to the concept of trust relationships between healthcare professionals at different hierarchical levels within hospital settings. The search strategy, as it is explained below in the Search strategy section, aimed to capture published literature that would answer several research questions. The first area the authors explored was what are the characteristics of trustworthy management in hospital settings [3]. The second area explored is the subject of the present systematic literature review.

The review is registered with PROSPERO under the following ID: CRD42023433021 and can be accessed on their website [26].

### Search strategy

Seven databases (Embase, MEDLINE, Web of Science, APA PsycInfo, Scopus, CINAHL and EconLit) and three publisher platforms (SAGE Journals, Taylor & Francis Online and Springer Link) were systematically searched to find eligible records for this review. These data sources were chosen because they cover relevant areas of research for the subject of this review, such as social sciences, nursing and allied health, medicine and healthcare policy and management. Moreover, the chosen databases such as Embase, MEDLINE and Web of Science cover a wide range of published records. The search strategy was developed in collaboration with a university librarian and over the course of a long and iterative process of running different versions and ample discussions among the authors as well as with experts on systematic reviews.

The initial search strategy has three components and has the following structure: 1) “hospital(s)” OR “ward(s)” AND 2) “health care professional(s)” OR “doctor(s)” OR “nurse(s)” OR “leader(s)” OR “manager(s)” NEAR/15 3) “trust” OR “reliance” OR “credibility”. The first component represents the setting (hospitals); the second component encompasses actors working in a hospital, such as healthcare staff (nurses, doctors etc.) and management staff. And the third component links the actors’ component to the focus of the project, namely trust relationships between these actors.

The search conducted in Taylor & Francis Online and SAGE Journals is limited to the use of the term “trust” only in the third component; as these platforms did not allow the use of wildcards. A wildcard is a character, such as * for example, and can be used to substitute one or more characters in a word in order to make the search string more efficient. This means, in the case of this review, that plurals and variations that would have been captured by wildcards had to be written separately, resulting in a very long search string. The search engine of these platforms allow for a certain length of a search string to be inputted; and although a complete search string was developed to include all terms, it exceeded the limit. Thus the search string used for these two platforms does not include the terms “reliance” and “credibility” under the third component.

For the modified search strategy, the two actor components were separately linked to the third component and connected with the AND operator, having the following structure: (“hospital(s)” OR “ward(s)”) AND (((“health care professional(s)” OR “doctor(s)” OR “nurse(s)”) NEAR/15 (“trust” OR “reliance” OR “credibility”)) AND ((“leader(s)” OR “manager(s)”) NEAR/15 (“trust” OR “reliance” OR “credibility”))).

The first run of the search strategy took place on 9th of August 2021. Given how much time had passed between the initial run and the start of working on the research question of this systematic review (October 2022); a second run was conducted, with time limits between August 2021 and 21st October 2022. The detailed search strategies of the first run from 9th of August, second run (August 2021 – 21st October 2022) and the run of the modified search strategy (August 2021 – 21st October 2022) can be found under Additional files 1, 2 and 3 respectively. No time limits were set for the initial run. For the consequent searches, where possible, time limits were set between August 2021 and 21st October 2022. Some databases only allowed years as time limits; and not months of the year. In these cases, time limits were set between 2021 and 2022. Where applicable, the searches were limited to English language.

### Eligibility criteria

Several inclusion criteria had to be met for studies to be included in the review. Firstly, studies had to research causes/determinants of or factors influencing trust relationships between colleagues or peers or team members, for example trust between nurses, between nurses and physicians, between physicians, and so on; and they had to be working in a hospital or a similar setting, such as wards where healthcare professionals are employed and patients receive treatment. We should note that nurses and physicians on the same place in the hierarchical structure are seen as colleagues in our study. In some countries physicians may be formally superior to nurses. Secondly, eligible studies had to be empirical with a qualitative, quantitative or mixed research design. And thirdly, they should be written in English. No time limit in terms of publication year was applied. No grey literature was included in this review since this type of material is not subject to transparent quality controls in the form of peer reviews.

A number of exclusion criteria were also imposed. Studies that were exploring trust relationships between providers on different formal hierarchical levels (such as trust between nurses and nurse managers, physicians and hospital managers, etc.) were excluded. This was done in order to capture published literature that focused on peer/coworker/colleague trust relationships and not supervisor/subordinate trust relationships. Moreover, papers with conceptually unclear research questions, setting, participants and trust relationships were excluded. Theoretical studies and systematic reviews were also excluded. Lastly, studies were excluded if they represented case studies that were anecdotal in nature, lacked a clear methodological framework and/or had a single informant.

### Record selection

**Fig. 1:**
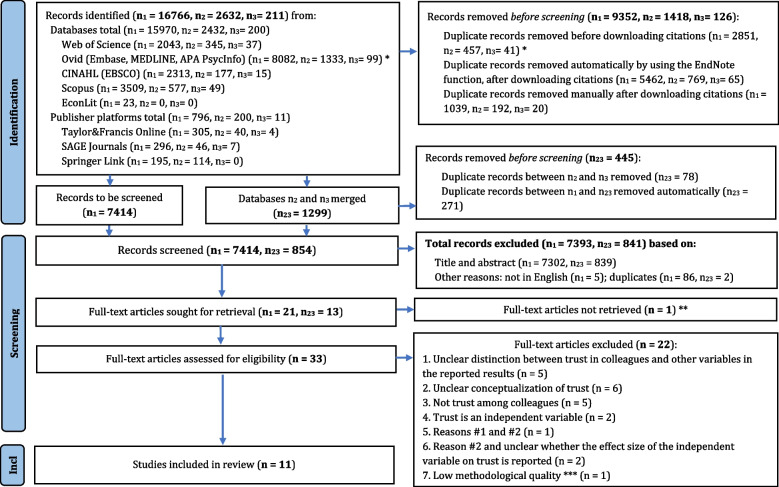
Flow diagram n1 = initial run of the search strategy (database inception – 09.08.2021); n_2_ = second run of the search strategy (August 2021 – 21.10.2022); n_3_ = running of the modified search strategy (August 2021 – 21.10.2022); n_23_ = database n_2_ joined with database n_3_; * = Duplicates from the records identified could be removed within the Ovid platform, before downloading the citations. ** Inaccessible. ***Article excluded after critical appraisal. Adapted from: Page, McKenzie [27]

Flow diagram Fig. 1 shows the identification, screening and inclusion processes. The initial search resulted in n_1_ = 16,766 records, the second run in n_2_ = 2632 records and the run of the modified search string in n_3_ = 211 records. A total of 10,896 duplicate records (n_1_ = 9352, n_2_ = 1418, n_3_ = 126) were removed before screening. Thus, from the initial search, a total of n_1_ = 7414 entered the screening phase. After duplicate removal, the second run database had n_2_ = 1214 records and the run of the modified search had n_3_ = 85 records. These two databases were then merged resulting in n_23_ = 1299 records. 78 duplicates were found and removed and n_23_ = 1221. This database was then checked for duplicates with n_1_ = 7414. The duplicates were found and removed automatically (271) and manually (96), resulting in a total of n_23_ = 854 records that entered the screening phase.

Both resulting databases were independently screened by at least two authors. The results of the first run (n_1_ = 7414) were screened by title and abstract for 2 research questions.The research question of this systematic review was one of them. Based on title and abstract, 7393 records did not meet the inclusion criteria; additionally 5 records were removed because they were not written in English and a further 86 duplicate records were identified and removed. After discussions regarding records that the authors were in disagreement on whether to include in the full-text review or not, n_1_ = 21 records were agreed upon to enter full-text revision from the initial search run.

The second database n_23_ = 854 was independently screened by at least two authors. 839 did not meet the inclusion criteria and 2 more duplicate records were found and removed. Disagreements over inclusion in full-text review were solved through discussions and n_23_ = 13 records were included in the full-text review.

Thus, 34 records (n_1_ = 21 and n_23_ = 13) were sought for retrieval. One record could not be retrieved, as no reply was given by the first author when the full-text request was sent through Research Gate. Thus, 33 records were retrieved in full-text and assessed for eligibility. The references of the 34 records were checked, but no additional records that met the eligibility criteria were found. All authors independently performed the full-text review and 12 papers passed the eligibility criteria. Of these, one record was excluded after the critical appraisal process due to its poor methodological quality and concerns regarding risk of bias. Thus, 11 papers were included in the review and the rest (*n* = 22) were removed based on the reasons listed in Fig. 1. The list of the excluded papers is available under Additional file 4.

### Data analysis and synthesis

A narrative synthesis technique was adopted to explore and discuss the causes/determinants/influencing factors of trust between colleagues identified across both quantitative and qualitative studies. This method “relies primarily on the use of words and text to summarise and explain the findings of the synthesis” [28]. We used tabulation as a tool to describe the studies included in the review and to “begin to identify patterns across studies” [28]. This data synthesis technique was deemed suitable since the review included studies with both quantitative and qualitative research designs and the data from the studies was not deemed appropriate for a meta-analysis or meta-ethnography.

The first author performed data extraction and another author checked for correctness. The following data items were extracted from each included study: author(s) and year of publication, aim, aspects related to theoretical / conceptual framework used, setting (e.g. hospital, clinic), participants of the study, measured concepts, type of data analysis, findings and country. Regarding findings, relevant results for the research question were extracted from the included studies. For quantitative studies, the data extracted related to hypothesis, results of the tests conducted and the strength of the relationship where reported; while from the included qualitative studies theme names, authors’ interpretation of supporting evidence and quotes from interviews were extracted. The detailed summary of included studies can be found under Additional file 5.

Once data extraction was complete, the authors of the review had multiple rounds of discussion relating to patterns identified within and across studies. More specifically, using the table that resulted from the data extraction process, under the findings column, factors that impacted trust were highlighted by bolding the words. Once these were highlighted, the identified factors were grouped in three categories, namely factors relating to professional competence, communication and ethics. The first two themes emerged from both the quantitative and qualitative included studies.

Each included study was critically appraised independently by at least two researchers. This was done to obtain an overview of the quality of the included studies and determine whether any papers should be excluded from the review due to their low quality which translates to a high risk for bias. The JBI Critical appraisal checklist for qualitative research [29] was used to assess the qualitative studies included in the review (*n* = 7). The rest (*n* = 5) had different quantitative research designs. Three of them were categorised as survey or questionnaire studies and these were appraised using a checklist by the National Institute for Health and Clinical Excellence (NICE) [30]. Response options were not given on this checklist, so in order to be consistent, the response options from the JBI checklist (Yes, No, Unclear and Not applicable) were used. One of the included quantitative studies was a longitudinal, quasi-experimental field study and was critically appraised using the JBI Critical Appraisal Checklist for Quasi-Experimental studies [31]. And lastly, there was also a cluster randomised controlled study included in the review that was assessed using the Revised Cochrane risk-of-bias tool for cluster-randomised trials (RoB 2 CRT) [32, 33]. This tool had its own overall appraisal scale while the rest did not. For those, a scale published by Roever [34] was used to rank the overall methodological quality of the included papers.

Table 1 shows the results of the critical appraisal conducted on the included records in this review. Of the 11 included papers, two papers were graded as high quality, two articles have a grading between acceptable and high, six papers are of acceptable quality and lastly, one article has a grading between acceptable and low quality.

**Table 1:** Critical appraisal of included studies

Author(s) (Year)	Quality Rating
Arluke [35]^a^/^b^	**Acceptable ( +) / Low (-)**
Calnan and Rowe [13]^a^	**Acceptable ( +)**
Campbell, Layne [36]^a^	**High (+ +)**
Eklof and Ahlborg [37]^c^	**Acceptable ( +)**
Kalisch, Russell [38]^d^	**Acceptable ( +)**
Luthans and Sommer [39]^e^	**High (+ +)**
McCabe and Sambrook [40]^a^	**Acceptable ( +) / High (+ +)**
Nakhaee and Nasiri [17]^a^	**Acceptable ( +) / High (+ +)**
Pawłowska [41]^a^	**Acceptable ( +)**
Tuan [42]^a^	**Acceptable ( +)**
Yoo, Zhang [43]^d^	**Acceptable ( +)**

^a^JBI critical appraisal checklist for qualitative research

^b^Checklist appraisal uncommon for the time

^c^Revised Cochrane risk-of-bias tool for cluster-randomised trials (RoB 2 CRT)

^d^NICE critical appraisal checklist for a questionnaire study

^e^JBI Critical Appraisal Checklist for Quasi-Experimental studies. (+ +) = high quality (majority of criteria met, little or no risk of bias); ( +) = acceptable (most criteria met, some flaws in the study with an associated risk of bias); (-) = low quality (either most criteria not met, or significant flaws relating to key aspects of study design) and (0) = reject (poor quality study with significant flaw, wrong study type, not relevant to guideline). Rating scale from Roever [34]

One quantitative, cross-sectional survey study [44] that was initially included in the review was graded as (0), poor quality and as a consequence was excluded. The main concerns for potential risk of bias arose from a poorly described Method section that was lacking information regarding the validity of the instruments used, the distribution and administration of the survey as well the response rate.

## Results

### Research methods, setting and participants, journals and countries

The summary of the included studies can be found in Table 2. Of the 11 included studies in the review, seven were qualitative studies that collected data through interviews (*n* = 3), focus groups (*n* = 1), field observations and interviews (*n* = 1) or a combination of the three (*n* = 2). The rest of the included studies (*n* = 4) had quantitative research designs. Two of the quantitative studies included were cross-sectional and collected the data at one point in time through a survey. One included study was a cluster randomised controlled study that collected data at baseline, 3-month and 6-month follow-up after the intervention. And the last quantitative study included was a longitudinal, quasi-experimental field study of an intervention where participants completed a survey at baseline, at mid-intervention 1 year from baseline and at the end of the intervention 2 years from baseline.

**Table 2:** Summary of included studies

Author(s) (Year)	Setting	Participants	Country	Findings
Arluke [35]^a^	Teaching hospital (*N* = 3)	Field observations and interviews (*N* = 68) with medical staff (interns, residents and attending physicians)	USA	During rounds, trust among peers is built by trying to stand out in front of an attending physician using "**competence and knowledge**", **but not at the expense of peers.** And by making peers "**look good during rounds**"
Calnan and Rowe [13]^a^	The primary care case study was carried out in a large multi-partner, training general practice and the secondary care case study in an orthopedic department in a teaching hospital	Face-to-face interviews with clinicians (N_1_ = 7, N_2_ = 16), managers (N_1_ = 2, N_2_ = 2) and patients (N_1_ = 10, N_2_ = 2)	UK	Mutual trust was seen as important, but conditional. Key dimensions were **competence, reliability, honesty**, and additionally for clinicians in the hospital setting, **confidentiality**. Trust could be **lost/earned** through **clinical interaction and communication**. J*unior doctors* consider trust in a more traditional way based on **seniority and medical hierarchy**
Campbell, Layne [36]^a^	Inpatient units (*N* = 2), chosen from 53 units based on the relational quality (RQ) score. The one with the highest and lowest relational quality score were included in this study	Each focus group (*N* = 2) comprised of registered nurses (RNs) (*n* = 3) and nursing assistants (NAs) (*n* = 3). Total number of participants *N* = 12	USA	Both groups (high and low RQ) report trust as crucial to the interaction. Trust is influenced by “accountability”, “effective conflict resolution”, "**collaborative teamwork**" and "**prioritizing patient needs**". In high RQ unit, participants used "**mindfulness and professionalism**" to build trust
Eklof and Ahlborg [37]^b^	Hospital units (*N* = 10)	Health care workers participated in a cluster randomised controlled study. Measurements took place at baseline (N_B_ = 345), 3-month (N_3_ = 280) and 6-month follow-up (N_6_ = 226)	Sweden	The effect analysis used the samples from 3-month and 6-month follow-up. The hypothesis concerning **the effect [of dialogue training] on trust/openness was not supported at ***p* **< 0.05**, although a positive tendency was observed. And the dialogue training intervention had positive and statistically significant impact on participative/psychological safety and social support
Kalisch, Russell [38]^b^	54 units across 4 hospitals	Direct care nursing providers such as registered nurses, licensed practical nurses and unlicensed personnel (*N* = 2265)	USA	**Average daily census** and **number of nursing assistants (NAs)** were negatively correlated with each of the five nursing teamwork subfactors (**trust** being one of them). The strength of the Pearson Correlation Coefficient for average daily census was *r* = 0.451 and for number of NA was *r* = 0.459, which the authors state it is a medium strength following Cohen’s guidelines
Luthans and Sommer [39]^b^	Medical rehabilitation hospital (*N* = 1)	Longitudinal, quasi-experimental field study of a downsizing intervention. Managers and employees completed a survey at T1 (baseline *N* = 296), T2 (mid intervention *N* = 261) and T3 (at the end of the downsizing process *N* = 291)	USA	Employees showed **lower workgroup trust** (although it did rebound) while managers reported higher levels of workgroup trust, after the **downsizing process**
McCabe and Sambrook [40]^a^	Hospitals (*N* = 2) (an acute and a community hospital)	Interviews with staff nurses (*N* = 28) and nurse managers (*N* = 11)	UK	Trust was mainly talked about and perceived as trustworthy management. However, **communication systems** are described as antecedents of trust among peers/colleagues. **Confidentiality and discretion** regarding resolving issues were linked to communication. If these are broken, trust is undermined. **Professionalism** was one of the attributes of trusted individuals and covers **efficiency, teamwork and support (peer support both from colleagues and managers). Professional competence, consistency, accountability and objectivity** were attributes of trusted line-managers and colleagues alike
Nakhaee and Nasiri [17]^a^	Educational hospitals (*N* = 2)	Physicians (*N* = 5) and nurses (*N* = 7) participated in unstructured interviewed	Iran	**Four themes were identified: “divergent attitudes, uneven distribution of power, mutual trust destructors, and prudence imposed on nurses”. Trust destructors** include: **Ethical shortcomings**: nurse perspective about physicians (attribute mistakes to nurses, arrogance); physician perspective (some nurses avoiding responsibility and disregarding others). **Competency weakness**: nurse perspective (professional principles replaced with financial motives); physician perspective (nurses failing to be up-to-date with scientific information, professional weakness)
Pawłowska [41]^a^	Hospital departments (*N* = 3)	Overt, multi-person and multiple observations. Unstructured interviews with hospital management representative, ward managers and nurses. Informal conversations with medical and non-medical personnel who did not have managerial functions	N/A^c^	Trust between nurses, physicians, and physicians and nursing staff “is higher when the relations between employees **move from a purely professional area to a social and friendly, or even just friendly, area.**” Author of the paper points out that the doctors in the study (both men and women) did not talk about the emotional states of nursing staff (who are lower in the medical hierarchy) and that this could result in a lower level of trust
Tuan [42]^a^	State-owned hospital (*N* = 1)	Data was collected through a case-study approach with hospital document collection, field observations and in-depth interviews (*N* = 51) with CEO, head doctors, doctors, head nurses and nurses	Vietnam	After clinical governance was in place, **clinical knowledge sharing** and **sustainable health-oriented values** (such as **building trust in patients together** and **cooperative research**) grew and formed knowledge- and identity-based trust
Yoo, Zhang [43]^b^	General hospital (*N* = 4)	Nurses (*N* = 230)	South Korea	**Clinical decision-making abilities** were found to correlate statistically significant and positive with trust. (*r* = 0.25 – weak positive linear correlation, *P* < 0.01). The pathway from **tacit knowledge sharing** to trust is positive and significant (β = 0.48, *P* < 0.01). The pathway from **explicit knowledge sharing** to trust is positive but statistically *insignificant (*β = 0.24, *P* = 0.19)

*N/A* Not Applicable

^a^Qualitative study

^b^Quantitative study

^c^No specific context provided/not clearly stated what country the study was conducted in. The author’s affiliation was with University of Lodz, Poland

Studies took place within hospitals (*n* = 5), hospital units or departments (*n* = 5) and a hospital department and a general practice (*n* = 1). Regarding participants, some studies focused on one healthcare professional category such as nurses (*n* = 3) or medical staff comprising of interns, residents and attending physicians (*n* = 1). One study investigated relationships between physicians and nurses, and one study explored the perspective of clinicians, managers and patients. Lastly, five studies included both employees with managerial functions (for example CEO, hospital manager, ward managers and nurse managers) and non-managerial employees such as staff nurses, doctors, administrative staff, counsellors, dieticians and therapists.

Four studies were conducted in the USA. Three studies took place in countries in Europe; the United Kingdom (*n* = 2) and Sweden (*n* = 1). The rest were conducted in Iran (*n* = 1), Vietnam (*n* = 1) and South Korea (*n* = 1). One study did not specifically state the country the study took place in [41]; however, the author was employed at a university in Poland.

Most studies (*n* = 8) were published within the last 10 years, one was published in 2008 and the rest (*n* = 2) before the year 2000 (1999 and 1980 respectively). Three studies were published in journals specifically related to the nursing discipline, while four papers were published in journals focused on the healthcare field more broadly. The remaining studies (*n* = 4) were published in non-medical journals.

### Conceptualization of trust

Table 3 presents how each included study conceptualized / measured trust.

**Table 3:** Conceptualization of trust in the included studies

Author(s) (Year)	Definition of trust
Arluke [35]^a^	No definition was provided, but the authors refer to trust among peers
Calnan and Rowe [13]^a^	No definition was provided, but the authors differentiate between clinician-patient trust, clinician-clinician trust and clinician-manager trust. The interviews were guided by topics related to the development of “new forms of trust relations in the NHS” and healthcare staff were first asked about their experiences at work; their roles and responsibilities and then the discussions were focused on themes related to trust
Campbell, Layne [36]^a^	No definition was provided, but one of the four interview questions was specifically about trust: “(Q3) What behaviours (from an RN or NA depending on the participant) help you build trust in that relationship?”
Eklof and Ahlborg [37]^b^	Trust was conceptualized as a communication-related factor and used to operationalize workplace communication. Trust /openness were measured using a situational outlook questionnaire developed by Ekvall [45] and Isaksen, Lauer [46] and was framed as “emotional safety in workplace relationships”
Kalisch, Russell [38]^b^	Mutual trust was conceptualized as the “belief that team members will act in ways that promote the aims of the team” and was measured using the Nursing Teamwork Survey developed by Kalisch, Lee [47], Salas, Sims [48]. However, the authors do not provide further information or examples of included items from the scale
Luthans and Sommer [39]^b^	Workgroup trust was measured using an instrument developed by Pearce, Sommer [49] and captured “the perception of shared objectives and mutual support”
McCabe and Sambrook [40]^a^	The authors acknowledge that the concept of trust is not easily defined and adopt the following definition for trust to be ‘‘one’s willingness to increase one’s vulnerability to another whose behaviour is not under one’s control’’ by Zand [50]. During the interviews, the participants were prompted to “describe and discuss trust” and were initially asked “What do you mean when you think about and talk about trust?” They were then prompted to talk about the “nature of trust between themselves, their colleagues and line-managers on their ward” as well as “trust within the wider organisations”. They were also asked about “factors contributing to trust”
Nakhaee and Nasiri [17]^a^	No definition was provided, but the interview question was “Can you describe your experience of working relationships with nurses/physicians in your workplace?” The authors then posed probing questions to capture the thoughts of the participants and provide clarity to their responses
Pawłowska [41]^a^	No definition was provided. The data was gathered through observations, unstructured interviews and informal conversations and the author used ethnography and the grounded theory methodology approaches
Tuan [42]^a^	The authors differentiate between calculus-, knowledge—and identity-based trust. Knowledge-based trust is understood as "trust grounded in knowledge about another party developed through repeated interactions " where the authors cite Holsapple and Wu [51]. While identity-based trust is "deemed to be a product of reciprocal understanding" where the authors cite Maguire, Phillips [52]. The interview discussions were around the issues of “clinical governance, leadership, organisational culture, knowledge sharing, and trust.”
Yoo, Zhang [43] ^b^	No definition was provided. Trust was measured using an instrument developed by McAllister [53] and translated by Lee [54]. However, the authors do not provide further information or examples of included items from the scale

*RN* Registered Nurse, *NA* Nursing Assistant

^a^Qualitative study

^b^Quantitative study

Six of the 11 included studies did not provide a definition of trust or a statement on how trust was conceptualized. Out of the six, five were qualitative and one was quantitative. One study, [35], did not provide information on how data was gathered and what questions guided the interviews, they simply referred to trust among peers. One study, [41] used ethnography and the grounded theory methodology approaches to gather data through observations, unstructured interviews and informal conversations and themes emerged from the data gathered. Two studies asked their participants about themes related to trust between clinicians [13] and what behaviours help build trust relationships between registered nurses and nursing assistants [36]; while one study broadly asked nurses and physicians about working relationships [17]. And the quantitative study [43] did not provide examples of included items from the scale used to measure trust among registered nurses.

The other five included studies provided to some degree a definition / conceptualization of trust. One quantitative study framed trust/openness as “emotional safety in workplace relationships” [37]. The other two quantitative studies conceptualized mutual trust as the “belief that team members will act in ways that promote the aims of the team” [38] and workgroup trust as “the perception of shared objectives and mutual support” [39]. One qualitative study [40] adopted a definition similar to the one which this review adopted, namely “one’s willingness to increase one’s vulnerability to another whose behaviour is not under one’s control’’ by Zand [50]. Lastly, one qualitative study framed trust under two categories, knowledge-based trust which builds through repeated interactions and identity-based trust which rises from mutual understanding [42].

### Factors influencing trust among colleagues

#### Findings from qualitative studies

Several factors that influence trust have been identified within the included qualitative studies. Two of the more predominant factors relate to professional competence and communication.

#### Professionalism

In Arluke´s study, trust among medical peers (interns and residents) could be formed by showcasing competence during rounds. However, if this was done at the expense of peers, trust could be undermined [35]. Similarly, in Calnan and Rowe’s study, trust among clinicians could be lost or earned by proving oneself competent, honest and reliable during clinical interactions and communication [13]. Professional competence and accountability were also attributes of trusted line-managers and colleagues alike for staff nurses in McCabe and Sambrook’s study [40].

Nurses in a high relational quality unit shared how they used “mindfulness and professionalism” to build trust [36]. Another attribute of trusted individuals brought forward by staff nurses and nurse managers was professionalism characterised by efficiency, teamwork and support both from colleagues and managers [40]. In contrast to this, trust between nurses and physicians in Pawlowska’s study was found to be stronger when these relationships shifted from purely professional to more social and friendly [41].

Competency weakness, on the other hand, was found to be a trust destructor for physicians and nurses in Nakhaee and Nasiri’s study [17]. Nurses lost trust in physicians when they replaced professional principles with financial motives. While physicians considered nurses’ competency weakness to stem from failing to be up-to-date with scientific information and professional weakness. The authors of this study, however, did not provide further clarifications to what they mean by professional weakness.

#### Communication

In Campbell et.al’s study, nurses from both high and low relational quality groups identified effective communication as an influencing factor of trust [36]. They also found that a work environment characterised by psychological safety fostered trust and promoted high relational quality. Similarly, McCabe and Sambrook described communication systems as antecedents of trust among nurses; where confidentiality and discretion when resolving issues were linked to communication; trust was lost without it [40]. On the same note, the clinicians in the hospital setting in Calnan and Rowe’s study consider confidentiality to be a key dimension of their trust relationships [13]. Lastly, another aspect of communication, namely clinical knowledge sharing, helped form knowledge- and identity-based trust among clinicians in Tuan’s study [42].

#### Ethics

Ethical shortcomings were found to hinder trust, e.g. in Nakhaee and Nasiri’s study. Nurses perceived physicians to be arrogant, and their trust relationship was undermined when physicians attributed their own mistakes to the nursing staff. From the physicians’ perspective, trust in nurses eroded when they avoid responsibility and disregard others [17]. This is in stark contrast with Tuan’s findings that shed light on how knowledge- and identity-based trust flourish when physicians and nurses share the same values, such as building trust in patients together and cooperative research [42].

### Findings from quantitative studies

#### Professionalism and communication

Similarly to the qualitative studies, which found that professional competence and communication influence trust, one quantitative study found that clinical decision-making abilities were weakly, but positively and significantly correlated with trust among nurses (*r* = 0.25, *P* < 0.01) [43]. And while the relationship between explicit knowledge sharing and trust was found to be positive but statistically insignificant (β = 0.24, *P* = 0.19); tacit knowledge sharing was found to positively and significantly influence trust (β = 0.48, *P* < 0.01) [43].

Eklof and Ahlborg [37] measured workplace communication “in the form of participative safety, trust/openness, and social support”. And the dialogue training intervention tested by them had a positive and statistically significant effect on participative /psychological safety and social support from supervisors. The hypothesis that the dialogue training would have an impact on trust/openness among healthcare workers was not significantly supported, but a positive tendency was observed. Nevertheless, trust is an important aspect of participative/psychological safety as definitions of psychological safety link the term to “mutual respect and trust among team members” [55].

In Kalisch, Russell and Lee’s study [38], nursing teamwork was measured using a survey that included several dimensions: participative safety, trust/openness, and social support. In their study, a negative relationship was observed between trust among nurses and average daily patient and nursing assistants (NA) census. This means that trust among nurses decreased when the number of patients and NAs in the unit increased.

#### Downsizing

Lastly, hospital employees showed lower workgroup trust than managers during a downsizing process that eventually rebound at the end of this process in Luthans and Sommer’s study [39].

## Discussion

### Professional competence

In the exploration of factors influencing trust within healthcare, professional competence was a critical theme that emerged across several of the studies. Specifically, the findings from Calnan and Rowe [13], Nakhaee and Nasiri [17], Arluke [35], McCabe and Sambrook [40] and Yoo, Zhang and Yun [43] underscore the role of competence, such as clinical decision-making abilities [43] and staying up-to-date with relevant scientific information [17], in cultivating or losing trust among healthcare professionals.

There are parallels between these and previous findings. In a review of the characteristics of trustworthy (and untrustworthy) management, we found that leaders’ competence (including medical competence and decision making skills) were related to being perceived trustworthy by staff [3]. Other studies have also found that clinical managers (especially physicians) seek to maintain and demonstrate their clinical skills and knowledge in order to gain legitimacy among staff and colleagues [56, 57].

Given the findings in our current study, and the parallel findings from existing literature, the role of competence could reflect the world view within Mintzberg's professional bureaucracy [58]. The professional bureaucracy is an organizational structure characterized by a high degree of specialization, where professionals operate with a significant level of autonomy, guided by standardized codes of practice and a shared body of knowledge. Within such an organizational structure, competence is not just an individual attribute but a systemic requirement that underpins the entire operational model. The professional bureaucracy relies on the competence of its members to function efficiently, where standardization of work processes is achieved through the adherence to professional norms and practices. This model may foster an environment where trust is built on demonstrated competence, allowing healthcare professionals to have confidence in each other's abilities and in their collective proficiency in addressing patient needs effectively.

### Communication and ethics

Communication was another prevalent factor from the included studies that had an impact on trust among colleagues. Effective communication as an overall construct was one of the influencing factors of trust that nurses talked about in Campbell et. al’s study [36]; while other studies [13, 40] highlighted how trust is conditioned by characteristics of communication, such as confidentiality, discretion and honesty. When these characteristics were broken, trust was undermined. Such findings have also been reported in other studies. For example, primary care providers and specialists built trust through repeated interactions and communication [59].

Another form of communication explored within one of the included studies is knowledge sharing, both tacit and explicit. However, only the pathway from tacit knowledge sharing to trust among nurses was positive and statistically significant [43]. Explicit knowledge is described by Nonaka and Takeuchi as information “expressed in words and numbers, and easily communicated and shared in the form of hard data, scientific formulae, codified procedures, or universal principles” [60]. Tacit knowledge is information that is hard to see or express, or “highly personal and hard to formalise, making it difficult to communicate or share with others” (ibid.).Thus, the results of the study conducted by Yoo, Zhang and Yun [43] included in this review highlight the significance of nurses deciding to share knowledge that was garnered through personal experiences for fostering trusting relationships with their colleagues.

Key characteristics of ethics described by Johnson and Ridley [61] could mean staying transparent, making oneself accountable, protecting confidential information and rectifying missteps immediately. Thus, trust between nurses and physicians was undermined when physicians failed to correct their mistakes, and instead deflected the responsibility of error on nurses, as shown in the study by Nakhaee and Nasiri [17]. In the same study, nurses who did not make themselves accountable by avoiding responsibility, lost the trust of physicians [17]. Accountability is also important in the findings of McCabe and Sambrook [40], where study participants considered accountability to be an attribute of trusted colleagues and line-managers. Moreover, the clinicians in Calnan and Rowe [13]’s study discussed honesty and confidentiality as key dimensions of mutual trust.

These findings are also reflected in other studies not included in our review. For example, a study on 187 Canadian human resources professionals revealed that perception of integrity, together with perception of ability and benevolence, were predictors of trust in peers [62]. Knoll and Gill [62] define integrity as “the trustor's perception that a trustee adheres to an acceptable set of ethical principles”.

Thus, encouraging healthcare professionals to display an ethical behavior in their day-to-day interactions with colleagues could lead to a flourishing trust relationship.

### Psychological safety and trust among colleagues in healthcare

Trust is an important enabler of good working environments and consequently also an enabler of high-quality patientcare [63, 64]. For many organisations, the big challenge is *how* to create trusting relationships at the workplace. One aspect of this challenge, which has received much and renewed attention in recent years, is the issue of psychological safety. The importance of this issue is raised in two of the articles which are part of this systematic review: Campbell, Layne and Scott [36] and Eklof and Ahlborg [37].

Schein and Bennis [65] refer to psychological safety as the extent to which individuals feel secure and confident in their ability to manage change. Kahn related psychological safety to individuals’ perceptions by defining it as “feeling able to show and employ one’s self without fear of negative consequences to self-image, status, or career” [66]. According to Kahn, people would feel safe in situations when they could trust that they will not suffer for personal engagement. A working environment characterised by psychological safety promotes trust, and vice versa. Edmondson [55] viewed psychological safety more as a collective entity, i.e. “a shared belief held by members of a team that the team is safe for interpersonal risk taking”. She linked situations of psychological safety to “mutual respect and trust among team members” (ibid.).

Based on a cluster randomised controlled study of approximately 300 Swedish healthcare workers, Eklof and Ahlborg [37] tested the effects of a dialogue training (DT) intervention on aspects of workplace communication relevant to teamwork and social support. They operationalized workplace communication as both participative safety and trust/openness. The former is characterised by factors such as the encouragement of interaction and sharing of information and views; the latter by, among other things, emotional security in workplace relationships, active management of conflicts, and freedom from explicitly negative communicative acts. Thus, both participative safety and trust/openness can be associated with psychological safety. The results from the study suggested that the dialogue training intervention had a significant positive effect on aspects of workplace communication related to participative safety, i.e. information sharing, mutual influence and a sense of having a common task.

Based on a qualitative analysis of two focus groups, Campbell, Layne and Scott [36] examined behaviours and experiences of registered nurses (RNs) and nursing assistants (NAs). RNs are important for providing safe care in a hospital environment, and the work relationship between RNs and NAs is critical to meet growing demands of inpatient care. Campbell, Layne and Scott [36] measured workplace communication in the form of participative safety, trust/openness, and social support. They found that among the important characteristics of the so-called “high relational quality group” was psychological safety, which included both individual perceptions of being encouraged to participate and speak freely, and mutual respect and trust between colleagues. In order to create trust, this group focused on mindfulness and professionalism. Thus, a work environment characterised by psychological safety was seen to foster trust and promote high relational quality among RNs and NAs.

### Quality of the included papers, conceptualization of trust and limitations

One qualitative paper [36] and one quantitative study with a longitudinal, quasi-experimental field study design [39] were graded as high quality papers with little or no risk of bias. McCabe and Sambrook [40]’s study and Nakhaee and Nasiri [17]’s study were graded between high and acceptable quality. For both these qualitative studies, an associated risk of bias arose from not addressing the influence of the researchers on the research and being unclear where the researchers conducting the study were located culturally or theoretically. This was also the case for the other qualitative studies rated as acceptable [13, 41, 42]. An additional risk of bias was associated with these studies due to failing to provide information regarding ethical approval. Lastly, the qualitative paper published in 1980 [35] was graded between acceptable and low due to the risk of bias stemming from all three aspects mentioned above (influence of researcher, locating researcher culturally or theoretically and ethical approval); as well as being unclear whether there is congruity between the methods used to collect data and research methodology. However, the absence of such information can be accounted for by the time period in which the paper was published.

Two of the three quantitative papers were rated as acceptable cross-sectional survey studies [38, 43], and an associated risk of bias arose from not discussing potential bias and being unclear whether the sampling frame was large enough and representative. While the third quantitative paper rated as acceptable was a cluster randomised controlled study [37] and there were some concerns for bias because participants might have been aware that they were in a trial and of the assigned intervention and because there was no pre-specified analysis plan.

Some risk of error could have happened given that only one author extracted the data and no standardised data extraction form was used. However, two other authors checked for correctness reducing the risk of error.

Regarding defining / conceptualizing trust within the included studies, six [13, 17, 35, 36, 41, 42] did not provide such a definition. However, for three of these studies, from how they framed the interview questions it can be argued that they refer to trust between colleagues in a general sense [13, 17, 36]. And for the five studies that did conceptualize trust [37–40, 42], there is some consistency across three. Namely, mutual trust was understood as team members’ notion that all will act towards promoting team aims [38]; workgroup trust was referred to as perceptions of “shared objectives and mutual support” [39] and in Tuan [42]’s study, trust was framed as knowledge-based trust built through repeated interactions and identity-based trust founded on mutual understanding, Overall, however, the absence of a definition for trust in some included studies and a degree of inconsistency across the ones that defined trust represents, on one hand, a limitation for this review. On the other hand, our review captured the heterogeneous nature of the concept itself by having identified different studies that operate with different definitions of trust. This finding highlighting that there is still a vast room for research on the topic of trust within healthcare organizations.

## Conclusion and future research

This study aimed to review the published literature to answer what factors influence the trust relationships between healthcare professionals working as colleagues in a hospital setting. We found that professional competence, clear communication, common ethics and psychological safety were factors that had a positive impact on these trust relationships.

Future studies could design and investigate interventions to increase trust relationship between healthcare professionals. Such interventions may focus on important aspects of a positive work environment, such as factors contributing to the enhancement of communication skills and feelings of psychological safety.

## Supplementary Information

Additional file 1. Initial run of the search strategy (09.08.2021).

Additional file 2. Second run of the search strategy (Aug 2021 – 21.10.2022).

Additional file 3. Modified search strategy (Aug 2021 – 21.10.2022).

Additional file 4. List of excluded papers and reasons.

Additional file 5. Detailed summary of included studies.

Additional file 6. JBI Checklist for Qualitative Research.

Additional file 7. NICE checklist for survey studies.

Additional file 8. JBI Checklist for Quasi-Experimental Appraisal Tool.

Additional file 9. Revised Cochrane risk-of-bias tool for cluster-randomized trials (RoB 2 CRT).

Additional file 10. PRISMA_2020_abstract_checklist.

Additional file 11. PRISMA_2020_checklist.

## Data Availability

All relevant data are within the paper and its attached Additional files.

## Abbreviations

CEO: Chief executive officer
DT: Dialogue training
EN: Enrolled nurse
JBI: Joanna Briggs Institute
NA: Nursing assistant
NICE: National Institute for Health and Clinical Excellence
RN: Registered nurse
RoB 2 CRT: Revised Cochrane risk-of-bias tool for cluster-randomised trials
RQ: Relational quality
UiO: University of Oslo
