# Supplementary material for: Factors influencing trust among colleagues in hospital settings: a systematic review
Source: BMC Health Serv Res. 2025 Jan 3;25:16. doi: 10.1186/s12913-024-12159-6 (PMC11697850; doi:10.1186/s12913-024-12159-6)
Supplement: Supplementary file 3 — Additional file 3. Modified search strategy (Aug 2021 – 21.10.2022). [file 12913_2024_12159_MOESM3_ESM.docx]

**Additional file 3**. Modified search strategy (August 2021 – 21.10.2022)

Modified search strategy

Initial search strategy

While performing the second run of the initial search strategy (which can be found in detail under Additional file 2); we became aware of the possibility to improve the initial search string by being more specific in linking the two different actor components with the trust component. The initial search strategy had a single actor component that combined the healthcare professionals level and the management level with the “OR” operator which was then linked to the trust component with a proximity operator (e.g. “NEAR”). In the modified search strategy, the actors components are linked individually to the trust component and then connected with the “AND” operator; modification that resulted in a lower number of hits.

|  | **Results of the first run of the modified search strategy (Aug 2021- 21.10.2022)** | |
| --- | --- | --- |
|  | Web of Science | 37 |
|  | Ovid (Embase, MEDLINE, APA PsycInfo) | 58 |
|  | CINAHL (EBSCO) | 15 |
|  | Taylor&Francis Online* | 4 |
|  | Springer Link | 0 |
|  | SAGE Journals* | 7 |
|  | Scopus | 49 |
|  | EconLit | 0 |
|  | Total (before duplicate removal) | 170 |
|  | Duplicates (EndNote) | 65 |
|  | Duplicates (manual) | 20 |
|  | **Total (after duplicate removal)** | **85** |

* Only “trust” included (search string limitation)

|  | **Web of Science** | | **10.08.2021 – 21.10.2022** | | | | |
| --- | --- | --- | --- | --- | --- | --- | --- |
| 1 | AB=(hospital$ OR ward$) OR TI=(hospital$ OR ward$) | | | | | | 128,694 |
| 2 | (AB=(((health NEAR/0 professional$) OR (healthcare NEAR/0 professional$) OR (health NEAR/0 care NEAR/0 professional$) OR (health NEAR/1 provider$) OR (healthcare NEAR/0 provider$) OR (health NEAR/1 care NEAR/1 provider$) OR physician$ OR doctor$ OR nurse$ OR (registered NEAR/0 nurse$) OR RN OR RNs OR (medical NEAR/1 doctor$) OR MD OR MDs OR (health NEAR/0 personnel) OR (medical NEAR/0 personnel) OR (health NEAR/0 care NEAR/0 personnel) OR (healthcare NEAR/0 personnel) OR (health NEAR/0 worker$) OR (healthcare NEAR/0 worker$) OR (health NEAR/0 care NEAR/0 worker$) OR (medical NEAR/2 staff) OR HCP OR HCPs OR (health NEAR/0 care NEAR/0 staff) OR (healthcare NEAR/0 staff) OR (health NEAR/0 staff)) NEAR/15 (trust* OR reliance OR credibility))) OR TI=(((health NEAR/0 professional$) OR (healthcare NEAR/0 professional$) OR (health NEAR/0 care NEAR/0 professional$) OR (health NEAR/1 provider$) OR (healthcare NEAR/0 provider$) OR (health NEAR/1 care NEAR/1 provider$) OR physician$ OR doctor$ OR nurse$ OR (registered NEAR/0 nurse$) OR RN OR RNs OR (medical NEAR/1 doctor$) OR MD OR MDs OR (health NEAR/0 personnel) OR (medical NEAR/0 personnel) OR (health NEAR/0 care NEAR/0 personnel) OR (healthcare NEAR/0 personnel) OR (health NEAR/0 worker$) OR (healthcare NEAR/0 worker$) OR (health NEAR/0 care NEAR/0 worker$) OR (medical NEAR/2 staff) OR HCP OR HCPs OR (health NEAR/0 care NEAR/0 staff) OR (healthcare NEAR/0 staff) OR (health NEAR/0 staff)) NEAR/15 (trust* OR reliance OR credibility)) | | | | | | 1,294 |
| 3 | (AB=((management OR manager$ OR (New NEAR/0 Public NEAR/0 Management) OR NPM OR leader$ OR leadership OR (governing NEAR/0 board$) OR (board NEAR/1 director$) OR (top NEAR/1 manage*) OR (middle NEAR/1 manage*) OR (chief NEAR/0 executive NEAR/0 officer) OR CEO OR supervisor$ OR governance OR governing) NEAR/15 (trust* OR reliance OR credibility))) OR TI=((management OR manager$ OR (New NEAR/0 Public NEAR/0 Management) OR NPM OR leader$ OR leadership OR (governing NEAR/0 board$) OR (board NEAR/1 director$) OR (top NEAR/1 manage*) OR (middle NEAR/1 manage*) OR (chief NEAR/0 executive NEAR/0 officer) OR CEO OR supervisor$ OR governance OR governing) NEAR/15 (trust* OR reliance OR credibility)) | | | | | | 2,689 |
| 4 | #2 AND #3 | | | | | | 107 |
| 5 | #1 AND #4 and English (Languages) | | | | | | 37 |
|  |  | | | | | |  |
|  | **Embase Classic+Embase**1947 to October 2022  **Ovid MEDLINE(R) ALL**1946 to October 2022  **APA PsycInfo**1806 to October 2022 | **2021 – Current 21.10.2022** | | | | | |
| 1 | (hospital? or ward?).ab,ti. | | | | | | 3,707,211 |
| 2 | limit 1 to english language | | | | | | 3,262,491 |
| 3 | limit 2 to yr="2021 - Current" | | | | | | 457,629 |
| 4 | (((health adj1 professional?) or (healthcare adj1 professional?) or (health adj1 care adj1 professional?) or (health adj2 provider?) or (healthcare adj1 provider?) or (health adj2 care adj2 provider?) or physician? or doctor? or nurse? or (registered adj1 nurse?) or RN or RNs or (medical adj2 doctor?) or MD or MDs or (health adj1 personnel) or (medical adj1 personnel) or (health adj1 care adj1 personnel) or (healthcare adj1 personnel) or (health adj1 worker?) or (healthcare adj1 worker?) or (health adj1 care adj1 worker?) or (medical adj3 staff) or HCP or HCPs or (health adj1 care adj1 staff) or (healthcare adj1 staff) or (health adj1 staff)) adj16 (trust* or reliance or credibility)).ab,ti. | | | | | | 28,420 |
| 5 | limit 4 to english language | | | | | | 27,000 |
| 6 | limit 5 to yr="2021 - Current" | | | | | | 4,364 |
| 7 | ((management or manager? or (New adj1 Public adj1 Management) or NPM or leader? or leadership or (governing adj1 board?) or (board adj2 director?) or (top adj2 manage*) or (middle adj2 manage*) or (chief adj1 executive adj1 officer) or CEO or supervisor? or governance or governing) adj16 (trust* or reliance or credibility)).ab,ti. | | | | | | 20,299 |
| 8 | limit 7 to english language | | | | | | 19,848 |
| 9 | limit 8 to yr="2021 -Current" | | | | | | 2,963 |
| 10 | 6 and 9 | | | | | | 339 |
| 11 | 3 and 10 | | | | | | 99 |
| 12 | remove duplicates from 11 | | | | | | 58 |
|  |  | | | | | |  |
| **CINAHL (EBSCO)** | | | | **Aug.2021 – 21.10.2022** | | | |
| 1 | TI ( hospital# OR ward# ) OR AB ( hospital# OR ward# ) **Limiters – English Language, Published Date: 20210801-** | | | | | | 41,41,713 |
| 2 | TI ( ((health N0 professional#) OR (healthcare N0 professional#) OR (health N0 care N0 professional#) OR (health N1 provider#) OR (healthcare N0 provider#) OR (health N1 care N1 provider#) OR physician# OR doctor# OR nurse# OR (registered N0 nurse#) OR RN OR RNs OR (medical N1 doctor#) OR MD OR MDs OR (health N0 personnel) OR (medical N0 personnel) OR (health N0 care N0 personnel) OR (healthcare N0 personnel) OR (health N0 worker#) OR (healthcare N0 worker#) OR (health N0 care N0 worker#) OR (medical N2 staff) OR HCP OR HCPs OR (health N0 care N0 staff) OR (healthcare N0 staff) OR (health N0 staff)) N15 (trust* OR reliance OR credibility) ) OR AB ( ((health N0 professional#) OR (healthcare N0 professional#) OR (health N0 care N0 professional#) OR (health N1 provider#) OR (healthcare N0 provider#) OR (health N1 care N1 provider#) OR physician# OR doctor# OR nurse# OR (registered N0 nurse#) OR RN OR RNs OR (medical N1 doctor#) OR MD OR MDs OR (health N0 personnel) OR (medical N0 personnel) OR (health N0 care N0 personnel) OR (healthcare N0 personnel) OR (health N0 worker#) OR (healthcare N0 worker#) OR (health N0 care N0 worker#) OR (medical N2 staff) OR HCP OR HCPs OR (health N0 care N0 staff) OR (healthcare N0 staff) OR (health N0 staff)) N15 (trust* OR reliance OR credibility) ) Published Date: 20210801-; Language: English | | | | | | 687 |
| 3 | TI ( (management OR manager# OR (New N0 Public N0 Management) OR NPM OR leader# OR leadership OR (governing N0 board#) OR (board N1 director#) OR (top N1 manage*) OR (middle N1 manage*) OR (chief N0 executive N0 officer) OR CEO OR supervisor? OR governance OR governing) N15 (trust* OR reliance OR credibility) ) OR AB ( (management OR manager# OR (New N0 Public N0 Management) OR NPM OR leader# OR leadership OR (governing N0 board#) OR (board N1 director#) OR (top N1 manage*) OR (middle N1 manage*) OR (chief N0 executive N0 officer) OR CEO OR supervisor? OR governance OR governing) N15 (trust* OR reliance OR credibility) ) Published Date: 20210801-; Language: English | | | | | | 323 |
| 4 | #2 AND #3 | | | | | | 61 |
| 5 | #1 AND #4 | | | | | | 15 |
|  |  | | | | | |  |
|  | **Taylor&Francis Online** | | | | | | **2021-2023** |
| 1 | [[All: hospital] OR [All: hospitals] OR [All: ward] OR [All: wards]] AND [[All: "health professional trust"] OR [All: "health professionals trust"] OR [All: "healthcare professional trust"] OR [All: "healthcare professionals trust"] OR [All: "health care professional trust"] OR [All: "health care professionals trust"] OR [All: "health provider trust"] OR [All: "health providers trust"] OR [All: "healthcare provider trust"] OR [All: "healthcare providers trust"] OR [All: "health care provider trust"]] AND [[All: "health care providers trust"] OR [All: "physician trust"] OR [All: "physicians trust"] OR [All: "doctor trust"] OR [All: "doctors trust"] OR [All: "nurse trust"] OR [All: "nurses trust"] OR [All: "registered nurse trust"] OR [All: "registered nurses trust"] OR [All: "rn trust"] OR [All: "rns trust"] OR [All: "medical doctor trust"] OR [All: "medical doctors trust"] OR [All: "md trust"] OR [All: "mds trust"] OR [All: "health personnel trust"] OR [All: "medical personnel trust~15"] OR [All: "health care personnel trust~15"] OR [All: "health care personnel trust~10"] OR [All: "health worker trust"] OR [All: "health workers trust"] OR [All: "healthcare worker trust"] OR [All: "healthcare workers trust"] OR [All: "health care worker trust"] OR [All: "health care workers trust"] OR [All: "medical staff trust"] OR [All: "hcp trust"] OR [All: "hcps trust"] OR [All: "healthcare staff trust"] OR [All: "health care staff trust"] OR [All: "health staff trust"]] AND [[All: "management trust"] OR [All: "manager trust"] OR [All: "managers trust"] OR [All: "new public management trust"] OR [All: "npm trust"] OR [All: "leader trust"] OR [All: "leaders trust"] OR [All: "leadership trust"] OR [All: "governing board trust"] OR [All: "governing boards trust"]] AND [[All: 15] OR [All: "board of directors trust"] OR [All: "top management trust"] OR [All: "top manager trust~15"] OR [All: "top managers trust~15"] OR [All: "middle management trust~15"] OR [All: "middle manager trust~15"] OR [All: "middle managers trust~15"] OR [All: "chief executive office trust"] OR [All: "ceo trust"] OR [All: "supervisor trust"] OR [All: "supervisors trust"] OR [All: "governance trust"] OR [All: "governing trust"]] AND [Publication Date: (01/01/2021 TO 31/12/2023)] | | | | | | 4 |
|  |  | | | | | |  |
|  | **Springer Link** | | | | | | **2021 - 2022** |
| 1 | (hospital OR hospitals OR ward OR wards) AND ((health NEAR/0 professional) OR (health NEAR/0 professionals) OR (healthcare NEAR/0 professional) OR (healthcare NEAR/0 professionals) OR (“health care” NEAR/0 professional) OR (“health care” NEAR/0 professionals)OR (health NEAR/1 provider) OR (health NEAR/1 providers) OR (healthcare NEAR/0 provider) OR (healthcare NEAR/0 providers) OR (“health care” NEAR/0 provider) OR (“health care” NEAR/1 providers) OR physician OR physicians OR doctor OR doctors OR nurse OR nurses OR (registered NEAR/0 nurse) OR (registered NEAR/0 nurses) OR RN OR RNs OR (medical NEAR/1 doctor) OR (medical NEAR/1 doctors) OR MD OR MDs OR (health NEAR/0 personnel) OR (medical NEAR/0 personnel) OR (“health care” NEAR/0 personnel) OR (healthcare NEAR/0 personnel) OR (health NEAR/0 worker) OR (health NEAR/0 workers) OR (healthcare NEAR/0 worker) OR (healthcare NEAR/0 workers) OR (“health care” NEAR/0 worker) OR (“health care” NEAR/0 workers)OR (medical NEAR/2 staff) OR HCP OR HCPs OR (“health care” NEAR/0 staff) OR (healthcare NEAR/0 staff) OR (health NEAR/0 staff)) NEAR/15 (trust* OR reliance OR credibility) AND (management OR manager OR managers OR “New Public Management” OR NPM​ OR leader OR leaders OR leadership OR (governing NEAR/0 board) OR (governing NEAR/0 boards) OR (board NEAR/1 directors) OR (top NEAR/1 manage*) OR (middle NEAR/1 manage*) OR “chief executive officer” OR CEO OR supervisor OR supervisors OR governance OR governing) NEAR/15 (trust* OR reliance OR credibility)) | | | | | | 0 |
|  |  | | | | | |  |
| **SAGE Journals** | | | | | **Aug 2021 - 2022** | | |
| 1 | (hospital OR hospitals OR ward OR wards) AND (“health professional trust”~15 OR “health professionals trust”~15 OR “healthcare professional trust”~15 OR “healthcare professionals trust”~15 OR “health care professional trust”~15 OR “health care professionals trust”~15 OR “health provider trust”~15 OR “health providers trust”~15 OR “healthcare provider trust”~15 OR “healthcare providers trust”~15 OR “health care provider trust”~15 “health care providers trust”~15 OR “physician trust”~15 OR “physicians trust”~15 OR “doctor trust”~15 OR “doctors trust”~15 OR “nurse trust”~15 OR “nurses trust”~15 OR “registered nurse trust”~15 OR “registered nurses trust”~15 OR “RN trust”~15 OR “RNs trust”~15 OR “medical doctor trust”~15 OR “medical doctors trust”~15 OR “MD trust”~15 OR “MDs trust”~15 OR “health personnel trust”~15 OR “medical personnel trust~15” OR “health care personnel trust~15” OR “health care personnel trust~10” OR “health worker trust”~15 OR “health workers trust”~15 OR “healthcare worker trust”~15 OR “healthcare workers trust”~15 OR “health care worker trust”~15 OR “health care workers trust”~15 OR “medical staff trust”~15 OR “HCP trust”~15 OR “HCPs trust”~15 OR “healthcare staff trust”~15 OR “health care staff trust”~15 OR “health staff trust”~15) AND (“management trust”~15 OR “manager trust”~15 OR “managers trust”~15 OR “New Public Management trust”~15 OR “NPM trust”~15 OR “leader trust”~15 OR “leaders trust”~15 OR “leadership trust”~15 OR “governing board trust”~15 OR “governing boards trust”15 OR “board of directors trust”~15 OR “top management trust”~15 OR “top manager trust~15” OR “top managers trust~15” OR “middle management trust~15” OR “middle manager trust~15” OR “middle managers trust~15” OR “chief executive office trust”~15 OR “CEO trust”~15 OR “supervisor trust”~15 OR “supervisors trust”~15 OR “governance trust”~15 OR “governing trust”~15) | | | | | | 7 |
|  |  | | | | | |  |
|  | **Scopus** | | | | | | **2021 - 2022** |
| 1 | TITLE-ABS ( hospital OR ward ) AND ( LIMIT-TO ( PUBYEAR , 2024 ) OR LIMIT-TO ( PUBYEAR , 2023 ) OR LIMIT-TO ( PUBYEAR , 2022 ) OR LIMIT-TO ( PUBYEAR , 2021 ) ) AND ( LIMIT-TO ( LANGUAGE , "English" ) ) | | | | | | 203,638 |
| 2 | TITLE-ABS ( ( ( health W/0 professional ) OR ( healthcare W/0 professional ) OR ( health W/0 care W/0 professional ) OR ( health W/1 provider ) OR ( healthcare W/0 provider ) OR ( health W/1 care W/1 provider ) OR physician OR doctor OR nurse OR ( registered W/0 nurse ) OR rn OR rns OR ( medical W/1 doctor ) OR md OR mds OR ( health W/0 personnel ) OR ( medical W/0 personnel ) OR ( health W/0 care W/0 personnel ) OR ( healthcare W/0 personnel ) OR ( health W/0 worker ) OR ( healthcare W/0 worker ) OR ( health W/0 care W/0 worker ) OR ( medical W/2 staff ) OR hcp OR hcps OR ( health W/0 care W/0 staff ) OR ( healthcare W/0 staff ) OR ( health W/0 staff ) ) W/15 ( trust* OR reliance OR credibility ) ) AND ( LIMIT-TO ( PUBYEAR , 2023 ) OR LIMIT-TO ( PUBYEAR , 2022 ) OR LIMIT-TO ( PUBYEAR , 2021 ) ) AND ( LIMIT-TO ( LANGUAGE , "English" ) ) | | | | | | 2,061 |
| 3 | TITLE-ABS ( ( management OR manager OR ( new W/0 public W/0 management ) OR npm OR leader OR leadership OR ( governing W/0 board ) OR ( board W/1 director ) OR ( top W/1 manage* ) OR ( middle W/1 manage* ) OR ( chief W/0 executive W/0 officer ) OR ceo OR supervisor? OR governance OR governing ) W/15 ( trust* OR reliance OR credibility ) ) AND ( LIMIT-TO ( PUBYEAR , 2023 ) OR LIMIT-TO ( PUBYEAR , 2022 ) OR LIMIT-TO ( PUBYEAR , 2021 ) ) AND ( LIMIT-TO ( LANGUAGE , "English" ) ) | | | | | | 4,774 |
| 4 | #2 AND #3 | | | | | | 158 |
| 5 | #1 AND #4 | | | | | | 49 |
|  |  | | | | | |  |
| **EconLit** | | | | | | **10.08.2021 - 2022** | |
| 1 | ti((hospital? OR ward?) AND (((health NEAR/0 professional?) OR (healthcare NEAR/0 professional?) OR (health NEAR/0 care NEAR/0 professional?) OR (health NEAR/1 provider?) OR (healthcare NEAR/0 provider?) OR (health NEAR/1 care NEAR/1 provider?) OR physician? OR doctor? OR nurse? OR (registered NEAR/0 nurse?) OR RN OR RNs OR (medical NEAR/1 doctor?) OR MD OR MDs OR (health NEAR/0 personnel) OR (medical NEAR/0 personnel) OR (health NEAR/0 care NEAR/0 personnel) OR (healthcare NEAR/0 personnel) OR (health NEAR/0 worker?) OR (healthcare NEAR/0 worker?) OR (health NEAR/0 care NEAR/0 worker?) OR (medical NEAR/2 staff) OR HCP OR HCPs OR (health NEAR/0 care NEAR/0 staff) OR (healthcare NEAR/0 staff) OR (health NEAR/0 staff) OR management OR manager? OR (New NEAR/0 Public NEAR/0 Management) OR NPM OR leader? OR leadership OR (governing NEAR/0 board?) OR (board NEAR/1 director?) OR (top NEAR/1 manage*) OR (middle NEAR/1 manage*) OR (chief NEAR/0 executive NEAR/0 officer) OR CEO OR supervisor? OR governance OR governing) NEAR/15 (trust* OR reliance OR credibility)) ) OR ab((hospital? OR ward?) AND (((health NEAR/0 professional?) OR (healthcare NEAR/0 professional?) OR (health NEAR/0 care NEAR/0 professional?) OR (health NEAR/1 provider?) OR (healthcare NEAR/0 provider?) OR (health NEAR/1 care NEAR/1 provider?) OR physician? OR doctor? OR nurse? OR (registered NEAR/0 nurse?) OR RN OR RNs OR (medical NEAR/1 doctor?) OR MD OR MDs OR (health NEAR/0 personnel) OR (medical NEAR/0 personnel) OR (health NEAR/0 care NEAR/0 personnel) OR (healthcare NEAR/0 personnel) OR (health NEAR/0 worker?) OR (healthcare NEAR/0 worker?) OR (health NEAR/0 care NEAR/0 worker?) OR (medical NEAR/2 staff) OR HCP OR HCPs OR (health NEAR/0 care NEAR/0 staff) OR (healthcare NEAR/0 staff) OR (health NEAR/0 staff) OR management OR manager? OR (New NEAR/0 Public NEAR/0 Management) OR NPM OR leader? OR leadership OR (governing NEAR/0 board?) OR (board NEAR/1 director?) OR (top NEAR/1 manage*) OR (middle NEAR/1 manage*) OR (chief NEAR/0 executive NEAR/0 officer) OR CEO OR supervisor? OR governance OR governing) NEAR/15 (trust* OR reliance OR credibility)) ) **Additional limits- Languages: English** | | | | | | 0 |
|  |  | | | | | |  |
