# Supplementary material for: Factors influencing trust among colleagues in hospital settings: a systematic review
Source: BMC Health Serv Res. 2025 Jan 3;25:16. doi: 10.1186/s12913-024-12159-6 (PMC11697850; doi:10.1186/s12913-024-12159-6)
Supplement: Supplementary file 4 — Additional file 4. List of excluded papers and reasons. [file 12913_2024_12159_MOESM4_ESM.docx]

| **Additional file 4.** List of excluded papers and reasons for exclusion | | | |
| --- | --- | --- | --- |
| **#** | **Author(s) (Year)** | **Reasons for exclusion** | **Comments** |
| 1 | Assegaai and Schneider (1) | Unclear distinction between trust in colleagues and other variables in the reported results | Not a clear distinction between results related to trust in colleagues and trust in managers |
| 2 | Ay and Oktay (2) | Low methodological quality | The Critical appraisal checklist for a questionnaire study by NICE (3) was used to assess the quality of this paper. The methods section is poorly described and lacks information regarding the validity of the instruments used, the distribution and administration of the survey, the response rate. This raised concerns for potential risk of bias and thus the authors excluded the paper from the review. |
| 3 | Berg and Hallberg (4) | Unclear conceptualization of trust | Trust among coworkers |
| 4 | Doos, Vinell (5) | Not trust among colleagues | Trust between nurse managers |
| 5 | Ferres, Connell (6) | Trust is an independent variable | Trust in peers |
| 6 | Grande, Berdida (7) | Unclear conceptualization of trust | Trust as a professional value |
| 7 | Gulzar, Hussain (8) | Unclear conceptualization of trust and unclear distinction between trust in colleagues and other variables in the reported results | Not a clear distinction between results related to trust in colleagues and trust in supervisors |
| 8 | Khan, Anwar Khan (9) | Unclear conceptualization of trust and unclear whether the effect size of the independent variable on trust is reported |  |
| 9 | Khan, Anwar Khan (10) | Unclear conceptualization of trust and unclear whether the effect size of the independent variable on trust is reported |  |
| 10 | Kuhlmann (11) | Unclear conceptualization of trust | The methods are not described. |
| 11 | Manojlovich, Harrod (12) | Unclear conceptualization of trust |  |
| 12 | McCabe and Sambrook (13) | Unclear distinction between trust in colleagues and other variables in the reported results | Not a clear distinction between results related to trust in colleagues and trust in line managers and immediate work environment |
| 13 | Norikoshi, Kobayashi (14) | Trust is an independent variable |  |
| 14 | Ramli, Noor (15) | Unclear conceptualization of trust |  |
| 15 | Smits, Boezeman (16) | Not trust among colleagues | Trust between nurses and family caregivers |
| 16 | Stasiulis, Gibson (17) | Unclear distinction between trust in colleagues and other variables in the reported results | Not a clear distinction between results related to trust among service providers and between service providers and management |
| 17 | Stasiulis (18) | Unclear distinction between trust in colleagues and other variables in the reported results | This is a dissertation that includes the other paper by Stasiulis (#15). No new data is reported. Not a clear distinction between results related to trust among service providers and between service providers and management |
| 18 | Tehranineshat and Torabizadeh (19) | Not trust among colleagues | Trust between nursing students and others (e.g. instructors, patients and family, nursing personnel). |
| 19 | Thude, Primdahl (20) | Not trust among colleagues | Trust in management |
| 20 | Vindrola-Padros, Ramsay (21) | Unclear distinction between trust in colleagues and other variables in the reported results | Not a clear distinction between results related to trust in colleagues and trust in leaders |
| 21 | von Behr, Cleaver (22) | Not trust among colleagues | Trust among non-clinical staff (operational, team-leader staff and top management staff). |
| 22 | Xu, Jia (23) | Unclear conceptualization of trust |  |

**References**

1. Assegaai T, Schneider H. Factors Associated With Workplace and Interpersonal Trust in the Supervisory System of a Community Health Worker Programme in a Rural South African District. International journal of health policy and management. 2021;24.

2. Ay FA, Oktay S. The Effect of Nepotism and Its Applications Leading to Ethical Collapse in Organizational Trust: A Research on Physicians and Nurses at a University Hospital. Turkish Journal of Business Ethics. 2020;13(1):159-67.

3. NICE. Sickle Cell Acute Painful Episode: Management of an Acute Painful Sickle Cell Episode in Hospital. Guidelines NIfHaCE, editor. Manchester (UK): National Institute for Health and Clinical Excellence (NICE)

Copyright © 2012, National Institute for Health and Clinical Excellence.; 2012.

4. Berg A, Hallberg IR. Effects of systematic clinical supervision on psychiatric nurses' sense of coherence, creativity, work-related strain, job satisfaction and view of the effects from clinical supervision: a pre-post test design. Journal of psychiatric and mental health nursing. 1999;6(5):371-81.

5. Doos M, Vinell H, von Knorring M. Going beyond "two-getherness": Nurse managers' experiences of working together in a leadership model where more than two share the same chair. Intensive & critical care nursing. 2017;43:39-46.

6. Ferres N, Connell J, Travaglione A. The effect of future redeployment on organizational trust. Strategic Change. 2005;14(2):77-91.

7. Grande RAN, Berdida DJE, Alshammari FF, Nacubuan OA, Alshammari TA, Alenezi HS, et al. Nurses' professional values and competency in Saudi Arabia: A structural equation modelling approach. Journal of clinical nursing. 2022;06.

8. Gulzar S, Hussain K, Akhlaq A, Abbas Z, Ghauri S. Exploring the psychological contract breach of nurses in healthcare: an exploratory study. Asia-Pacific Journal of Business Administration. 2022.

9. Khan HGA, Anwar Khan M, Ali MI, Khattak SI, Shujaat S, Alam BF. Trust, performance and level of happiness of healthcare employees in the presence of authentic leadership. Work. 2022;05.

10. Khan HGA, Anwar Khan M, Iftikhar Ali M, Salem S, Rashid S, Zahur H. Does authentic leadership influences performance of individuals in presence of trust and leader member exchange: an evidence from health care sector. Cogent Business and Management. 2022;9(1).

11. Kuhlmann E. Traces of Doubt and Sources of Trust:Health Professions in an Uncertain Society. Current Sociology. 2006;54(4):607-20.

12. Manojlovich M, Harrod M, Hofer T, Lafferty M, McBratnie M, Krein SL. Factors influencing physician responsiveness to nurse-initiated communication: A qualitative study. BMJ Quality and Safety. 2020.

13. McCabe TJ, Sambrook SA. A discourse analysis of managerialism and trust amongst nursing professionals. Irish Journal of Management. 2019;38(1):38-53.

14. Norikoshi K, Kobayashi T, Tabuchi K. A qualitative study on the attributes of nurses' workplace social capital in Japan. Journal of nursing management. 2018;26(1):74-81.

15. Ramli NR, Noor H, Zolkefli Y. Resilience among nurses working in paediatric wards in Brunei Darussalam: A qualitative study. Belitung Nursing Journal. 2021;7(6):535-41.

16. Smits MAA, Boezeman EJ, Nieuwenhuijsen K, De Boer A, Van Dijkum E, Eskes AM. Family involvement on nursing wards and the role conflicts experienced by surgical nurses: A multicentre cross-sectional study. Scandinavian Journal of Caring Sciences. 2022;36(3):706-16.

17. Stasiulis E, Gibson BE, Webster F, Boydell KM. Resisting governance and the production of trust in early psychosis intervention. Social Science & Medicine. 2020;253.

18. Stasiulis EK. The work of getting better: An institutional ethnography of an early psychosis intervention clinic. Dissertation Abstracts International: Section B: The Sciences and Engineering. 2022;83(10-B):No Pagination Specified.

19. Tehranineshat B, Torabizadeh C. Dignity of Nursing Students in Clinical Learning Environments. Nursing Ethics. 2022;29(3):742-57.

20. Thude BR, Primdahl J, Jensen HI, Elkjaer M, Hoffmann E, Boye LK, et al. How did nurses cope with the fast, comprehensive organisational changes at Danish hospital wards during the COVID-19 pandemic? An interview study based on nurses' experiences. Bmj Open. 2021;11(12).

21. Vindrola-Padros C, Ramsay AI, Black G, Barod R, Hines J, Mughal M, et al. Inter-organisational collaboration enabling care delivery in a specialist cancer surgery provider network: A qualitative study. Journal of Health Services Research & Policy. 2022;27(3):211–21.

22. von Behr CM, Cleaver I, Minshall T, Clarkson PJ. Trust and knowledge sharing among hospitals during COVID-19: the compound effect of four barriers to organisational trust for knowledge sharing. VINE Journal of Information and Knowledge Management Systems. 2022.

23. Xu X, Jia S, Zhang S, Mai X, Mao Z, Han B. Analysis of the factors influencing teamwork among oncology nurses based on multigroup structural equation model. Annals of Translational Medicine. 2022;10(18) (no pagination).
