# Supplementary material for: Factors influencing trust among colleagues in hospital settings: a systematic review
Source: BMC Health Serv Res. 2025 Jan 3;25:16. doi: 10.1186/s12913-024-12159-6 (PMC11697850; doi:10.1186/s12913-024-12159-6)
Supplement: Supplementary file 5 — Additional file 5. Detailed summary of included studies. [file 12913_2024_12159_MOESM5_ESM.docx]

| **Additional file 5**. Detailed summary of included studies | | | | | | | | |
| --- | --- | --- | --- | --- | --- | --- | --- | --- |
| **Author(s)** | **Aim** | **Setting** | **Theoretical/**  **conceptual framework** | **Participants** | **Measured concepts** | **Data analysis** | **Findings** | **Country** |
| Arluke (1) | To describe the operation of an inherent control mechanism (roundsmanship) | Teaching hospital (N=3) | N/A | Field observations and interviews (N=68) with medical staff (interns, residents and attending physicians) | The non-participant observations were conducted by the author of the paper over the course of 18 months; where he attended all the rounds that took place on the medical teaching wards. No interview guide was provided in the paper, but both interviews and observations were “directed by a general concern with the process and problems of social control in the context of professional training”. | Not stated | Roundsmanship was used by physicians in training to establish trust among peers on the ward by demonstrating “competence and knowledge” to stand out “in front of an attending physician, but not at the expense of a fellow trainee”; and by “making a fellow trainee look good” during the rounds. | USA |
| Calnan and Rowe (2) | To “explore how trust between clinicians and managers based in an acute setting involving close working in multi-disciplinary teams might differ from trust relations in a primary care setting where GPs and other practice staff have traditionally worked more autonomously.” | The primary care case study was carried out in a large multi-partner, training general practice and the secondary care case study in an orthopaedic department in a teaching hospital. | Based on the analysis of theoretical, policy and empirical literature, the authors developed a conceptual framework that proposes how trust and trust relations in the NHS may have changed. It addressed patient-clinician, clinician-clinician and clinician-manager relationships. In terms of clinician-clinician, their framework, postulated that there is a shift from “peer” trust based on seniority towards “earned” trust. | Face-to-face interviews with clinicians (N_1_=7, N_2_=16), managers (N_1_=2, N_2_=2) and patients (N_1_=10, N_2_=2). | To allow respondents to describe their experiences, spontaneous talk was emphasized during the interviews; and they were guided by topics related to the development of “new forms of trust relations in the NHS”. Topic guides were customized for each category of participants (patients, clinicians and managers). Healthcare staff were first asked about their experiences at work and their roles and responsibilities; and then the discussions were focused on themes related to trust. | Thematic inductive analysis and analysis of individual case histories. | Regarding clinicians, from the discussions with them it became apparent that mutual trust is very important. Key dimensions to this relationship were competence, reliability and honesty. It was also revealed that trust was described as conditional and could be earned or lost “through clinical interactions and communication” which posed as opportunities to showcase competence, honesty and reliability. The differences noted between the two cases were that in the acute setting, confidentiality was deemed quite important. Some health care staff, such as nurses assistants in primary care and junior doctors in secondary care, thought of trust in a more traditional way, where senior staff were considered trustworthy due to “their seniority and place in the medical hierarchy”. | UK |
| Campbell, Layne (3) | “To explore [registered nurses] (RNs) and [nursing assistants] (NAs) behaviours and experiences that promote patient safety and teamwork and enhance communication between RNs and NAs within the hospital environment” | Inpatient units (N=2), chosen from 53 units based on the relational quality (RQ) score.  This was measured in a previous study. The one with the highest and the one with the lowest relational quality score were included in this study. | N/A - short introduction and no conceptual or theoretical framework described. | Each focus group (n=2) comprised of RNs (n=3) and NAs (n=3). Total number of participants n=12. | The semi-structured interview guide had four questions and one was specifically about trust: “(Q3) What behaviors (from an RN or NA depending on the participant) help you build trust in that relationship?” | Inductive content analysis | The participants from both groups (high and low RQ) reported that trust was one of the crucial components to influence the interaction between RN and NA. And that in turn, trust was influenced by “effective communication, collaborative teamwork, and prioritizing patient needs”. Participants from the high RQ unit used mindfulness and professionalism to build trust. A RN on examples of trust: “I would say communication. Being open and [having an open] tone. How you approach someone, how do you talk with them? It has a professional level but also an assertive level.”. | USA |
| Eklof and Ahlborg (4) | To test the effects of a dialog training (DT) intervention on aspects of workplace communication relevant to teamwork, and social support in hospital units. | Trust/openness is one of the two communication-related factors used to operationalize workplace communication. It refers to “emotional security in workplace relationships, openness in communication, active management of conflicts, interpersonal trust and freedom from explicitly negative communication acts” – from Ekvall 1996 | Hospital units (N=10) | Health care workers participated in a cluster randomized controlled study. Measurements took place at baseline (N_B_=345), 3-month follow-up (N_3_=280) and 6-month follow-up (N_6_=226). | Participative safety, trust/openness and social support. | Effect analyses used two-sided Mann-Whitney U-tests. | The effect analysis used the samples from 3-month and 6-month follow-up. The hypothesis concerning the effect [of dialog training] on trust /openness was not supported at p<0.05, although a positive tendency was observed. | Sweden |
| Kalisch, Russell (5) | “To determine the relationship between the team and unit size and level of nursing teamwork” | The study is based on a modified version of Salas et al.s conceptual framework that explains “core components and coordinating functions of teamwork”. The modifications are based on a previous study by Kalisch, Lee and Salas which yielded 5 of the 8 elements from Salas’ theory: (a) team orientation, (b) team leadership, (c) backup, (d) shared mental models and (e) mutual trust – “belief that team members will act in ways that promote the aims of the team”. | 54 units across 4 hospitals | Direct care nursing providers (N=2265) | Nursing teamwork from which 5 factors emerged from the exploratory and confirmatory factor analyses (trust, team orientation, backup, shared mental model and team leadership); unit size (number of registered nurses, number of nursing assistants, number of total staff and average daily census). | Descriptive statistics, Pearson’s correlation and one-way analysis of variance. | Average daily census and number of nursing assistants were negatively correlated with each of the five nursing teamwork subfactors (trust being one of them). The strength of the Pearson Correlation Coefficient for average daily census was r=0.451 and for no. of NA was r=0.459, which the authors state it is a medium strength following Cohen’s guidelines. | USA |
| Luthans and Sommer (6) | “To make a contribution to the […] knowledge on the effects that downsizing has on the attitudes of health care managers and front-line employees”. Results are reported on the sample as a whole, but also separately (managers/staff) | The authors present an extensive literature review as a foundation for their study. It first explores “relevant literature on downsizing” – specifically focusing on downsizing strategies and their consequences on the organizational members, particularly on those surviving the downsizing. Secondly, literatures on downsizing in the field of health care is examined. | Medical rehabilitation hospital (N=1) | Longitudinal, quasi-experimental field study of a downsizing intervention. Managers and employees completed a survey at T1 (baseline N=296), T2 (mid intervention N=261) and T3 (at the end of the downsizing process N=291). | Organizational commitment, job satisfaction, supervisor support, workgroup trust (which measured the perception of shared objectives and mutual support). | Descriptive statistics, intercorrelations, multiple regression analysis, multiple ANCOVAs with repeated measures, univariate ANOVA tests | Workgroup trust for the whole sample (and other attitudes) significantly declined over the downsizing intervention. Employees showed lower workgroup trust (although it did rebound) while managers reported higher levels of workgroup trust. | USA |
| McCabe and Sambrook (7) | “To explore the antecedents, attributes and consequences of the concept of trust amongst nursing professionals, at individual, interpersonal and organisational levels.” | Hospitals (N=2) (an acute and a community hospital). | Having as a starting point a concept analysis framework within the nursing field drawn out by Walker and Avant 1988 and Rodgers 1989, the authors review the theoretical relationship between antecedents, attributes and consequences of trust and analyse these among nurses and nurse managers. The background section is extensive and describes in detail the existing theories on the subject. | Interviews with staff nurses (N=28) and nurse managers (N=11) | Semi-structured interview guide was informed by a comprehensive literature review and piloted among 4 nurses. It included open-ended questions on: “(1) how the participants conceptualized trust and the level of trust within their working environment; (2) the characteristics and attributes of trust and trustworthy managers; (3) the consequences of low trust.” Each participant was prompted to “describe and discuss trust” and were initially asked “What do you mean when you think about and talk about trust?”. They were then prompted to talk about the nature of trust between themselves, their colleagues and line-managers on their ward” as well as “trust within the wider organisations”. They were also asked about “factors contributing to trust”. | Concept analysis framework – thematic analysis that was guided by the theoretical relationship described in the background section. | The findings show that nurses’ discourses and perceptions on the concept of trust mainly focus on characteristics of trustworthy managers.  Findings on antecedents of trust that describe this relationship among peers/colleagues relate to communication systems.  Confidentiality and discretion was one theme linked to communication. The participants “stressed the importance of knowing that they could confined in their colleagues and managers in resolving any problems or issues they were experiencing”; and that if this confidentiality would be broken /compromised, their trust levels would be undermined.  Professionalism was one of the attributes of trusted individuals and this theme encompasses efficiency, teamwork and support. Peer support and teamwork from both colleagues and managers was considered “very important in developing trust.” For example, a nurse from the acute hospital stated that: “You have to rely on other people to help. You can’t do it all by yourself. You have to trust your colleagues, their work and their abilities (AN5)”. Moreover, "‘professional competence’,‘consistency’, ‘accountability’ and ‘objectivity’ in decision-making and behaviour were viewed as attributes of ‘trusted’ line-managers and colleagues."  Another nurse from the acute hospital said that: “Its whether or not you have faith in that person’s  ability to do their job. Whether you like the individual concerned is not important. You can actually dislike someone intensely, but at the same time you can trust and work with them so it is not personal (AN3)”.  Both nurses and nurse managers talked about the challenges of building a trust relationship with “agency nurses”, nurses on short-term contract and untrained nursing staff that arose from not having enough “knowledge of their qualities and attributes” and not enough time “to get to know them”. | UK |
| Nakhaee and Nasiri (8) | To “explore the major issues of nurse-physician inter-professional relationships in Iran.” | Educational hospitals (N=2) | N/A | Physicians (N=5) and nurses (N=7) participated in unstructured interviewed | The interviews were unstructured and they started with an open-ended question: “Can you describe you experience of working relationships with nurses/physicians in your workplace?”. To follow participants’ thoughts more exactly and to clarify responses during the interview, probing questions were asked. | Conventional content analysis methods. | Ethical shortcomings (“attribution of mistakes to the nurses [by physicians for their own mistakes], some physicians’ arrogance, shirking responsibility by some nurses and nurse’s disregard for others”) and competency weaknesses (“nurses’ negligence of up-to-date information and professional weakness, replacing professional principles with financial motivations [from nurses’ perspective about physicians]) were identified as mutual trust destructors. | Iran |
| Pawłowska (9) | “To show how an individually-determined level of power and status can shape relations between employees, their methods of communication, and emotions in the work-place.” | Hospital departments (N=3) | The theoretical framework of the sociology of emotions (the structural-interactive perspective) by Theodore Kemper is used as a base for the analysis conducted in this study. A detailed background describes the theoretical considerations. | Overt, multi-person and multiple observations. Unstructured interviews with hospital management representative, ward managers and ward nurses. Informal conversations with medical and non-medical personnel who did not have managerial functions. | N/A – unstructured interviews and conversations | Data analysis derived from grounded theory methodology: coding (open/factual, selective and theoretical); preparation of memos, theoretical sampling, matrix of conditions and focused category coding. | The findings are grouped under three categories: “ “disrespect game,” ” holding emotions,” and pride.  None of the interviewed physicians “rationalized the behaviour and emotions of staff who are lower” in the hierarchy than their own position (such as nursing staff). This lowering of status of nurses can lead to lower levels of trust between physicians and nursing staff. Trust between nurses, physicians, and physicians and nursing staff “is higher when the relations between employees move from a purely professional area to a social and friendly, or even just friendly, area.”  For example, nursing staff in the intensive care unit talked about positive relations between colleagues more often than staff in other units. | ? |
| Tuan (10) | “To look through the data of [a state-owned hospital] for evidence on whether a clinical governance initiative cultivates ethical leadership, market-or innovation-oriented culture, knowledge sharing, knowledge- or identity-based trust.” | State-owned hospital (N=1) | The literature review section is detailed and presents the concepts of *clinical governance*, *organisational culture*, *leadership*, *knowledge sharing* and *trust*. Trust is conceptualized as three stages/types: (1) calculus-based (cited here are Lewicki and Bunker 1995); (2) knowledge-based trust (cited here are Holsapple and Wu 2008); and (3) identity-based trust (cited here are Maguire et.al 2001, Lewicki and Bunker 1995 and Atwater 1988). The authors also have built a theoretical framework on several propositions and one of them relates to trust: “P4. Clinical governance initiative promotes knowledge- or identity-based trust.” | Data was collected through a case-study approach with hospital document collection, field observations and in-depth interviews (N=51) with CEO, head doctors, doctors, head nurses and nurses. | Interviews started with interviewees introducing themselves, depicting the hospital and its history and the nature of operations. Then there were discussion around the issues of: “clinical governance, leadership, organisational culture, knowledge sharing, and trust.” | Data was analysed by pattern-matching and explanation-building methods. | “Once the clinical governance mechanism was in place […] the sharing of clinical knowledge as well as sustainable health-oriented values also grew, leading to the formation of knowledge- and identity-based trust among clinicians.”  In terms of knowledge sharing, information picked up by nurses during patient observations and verbal interaction when providing care to the patients was then shared with physicians and it helped give more accurate diagnosis, treatment and prognosis.  Physicians and nurses shared the value of “building trust in patients together”; which lead to fostering identity-based trust between themselves as well. A nurses stated: “A teen patient had a seizure when I started to give her an injection of antibiotic. Dr T. came  and stayed by her until her seizure and apprehension vanished. He himself then gave the little patient an injection. He also stayed by her for me to give the next injection in the afternoon.  We together, through cooperation, created patient confidence. We together created this value and shared this value (Nurse, Department of Pediatrics).”  Cooperative research was another shared value that “reinforces identity-based trust”. One of the quotes provided states: “Prior to the implementation of clinical governance initiative, the notion of scientific research only referred to individual works by university doctors. Clinicians, especially nurses, did not  think of a role in a scientific research. Clinical governance encourages every clinician to participate in research. We nurses help doctors and professors to collect specimens, record changes in patients’ vital signs, etc. as a part of research. We together published our studies  and applied their findings in treatment and nursing practice (Nurse, Department of Neurology and Hematology).” | Vietnam |
| Yoo, Zhang (11) | “To investigate the effects of explicit and tacit knowledge sharing on clinical decision-making abilities and the mediating role of trust among registered nurses.” | The authors present a literature review on knowledge sharing (tacit and explicit) as a basis for their study. | General hospital (N=4) | Nurses (N=230) | Knowledge-sharing behavior (explicit and tacit knowledge-sharing), trust, clinical decision-making abilities (with 4 subscales). | Pearson’s  correlation, path analysis using SEM. | Clinical decision-making abilities were found to correlate statistically significant and positive with trust. (r=0.25 – weak positive linear correlation).  The pathway from tacit knowledge sharing to trust is positive and significant (β= 0.48, P < 0.01).  The pathway from explicit knowledge sharing to trust is positive but statistically insignificant. | South Korea |

**References**

1. Arluke A. Roundsmanship: Inherent control on a medical teaching ward. Social Science and Medicine. 1980;14 A(4):297-302.

2. Calnan M, Rowe R. Trust relations in a changing health service. Journal of Health Services Research & Policy. 2008;13(3_suppl):97-103.

3. Campbell A, Layne D, Scott E. Relational Quality of Registered Nurses and Nursing Assistants: Influence on Patient Safety Culture. Healthcare. 2021;9(2).

4. Eklof M, Ahlborg GA. Improving communication among healthcare workers: a controlled study. Journal of Workplace Learning. 2016;28(2):81-96.

5. Kalisch BJ, Russell K, Lee KH. Nursing Teamwork and Unit Size. Western Journal of Nursing Research. 2013;35(2):214-25.

6. Luthans BC, Sommer SM. The impact of downsizing on workplace attitudes: Differing reactions of managers and staff in a health care organization. Group & Organization Management. 1999;24(1):46-70.

7. McCabe TJ, Sambrook S. The antecedents, attributes and consequences of trust among nurses and nurse managers: A concept analysis. International Journal of Nursing Studies. 2014;51(5):815-27.

8. Nakhaee S, Nasiri A. Inter-professional Relationships Issues among Iranian Nurses and Physicians: A Qualitative Study. Iranian Journal of Nursing and Midwifery Research. 2017;22(1):8-13.

9. Pawłowska B. Emotions of Medical Personnel versus the Status and Power at Work in Hospital Wards. Przeglad Socjologii Jakosciowej. 2021;17(4):68-87.

10. Tuan LT. Clinical governance: A lever for change in Nhan Dan Gia Dinh Hospital in Vietnam. Clinical Governance. 2012;17(3):223-47.

11. Yoo KH, Zhang YA, Yun EK. Registered Nurses (RNs)' knowledge sharing and decision-making: the mediating role of organizational trust. International Nursing Review. 2019;66(2):234-41.
