# Supplementary material for: Factors influencing trust among colleagues in hospital settings: a systematic review
Source: BMC Health Serv Res. 2025 Jan 3;25:16. doi: 10.1186/s12913-024-12159-6 (PMC11697850; doi:10.1186/s12913-024-12159-6)
Supplement: Supplementary file 7 — Additional file 7. NICE checklist for survey studies. [file 12913_2024_12159_MOESM7_ESM.docx]

| ***Critical appraisal checklist for a questionnaire study***  **Research question and study design** | Yes | No | Unclear | Not applicable |
| --- | --- | --- | --- | --- |
| Was a questionnaire the most appropriate method? | □ | □ | □ | □ |
| **Validity and reliability** |  |  |  |  |
| Have claims for validity been made, and are they justified?  (Is there evidence that the instrument measures what it sets out to measure?) | □ | □ | □ | □ |
| Have claims for reliability been made, and are they justified? (Is there evidence that the questionnaire provides stable responses over time and between researchers?) | □ | □ | □ | □ |
| **Format** |  |  |  |  |
| Are example questions provided? | □ | □ | □ | □ |
| Did the questions make sense, and could the participants in the sample understand them? Were any questions ambiguous or overly complicated? | □ | □ | □ | □ |
| **Piloting** | □ | □ | □ | □ |
| Are details given about the piloting undertaken | □ | □ | □ | □ |
| Was the questionnaire adequately piloted in terms of the method and means of administration, on people who were representative of the study population? | □ | □ | □ | □ |
| **Sampling** |  |  |  |  |
| Was the sampling frame for the definitive study sufficiently large and representative? | □ | □ | □ | □ |
| **Distribution, administration and response** | □ | □ | □ | □ |
| Was the method of distribution and administration reported | □ | □ | □ | □ |
| Were the response rates reported, including details of participants who were unsuitable for the research or refused to take part? | □ | □ | □ | □ |
| Have any potential response biases been discussed? | □ | □ | □ | □ |
| **Coding and analysis** |  |  |  |  |
| What sort of analysis was carried out and was this appropriate? (e.g. correct statistical tests for quantitative answers, qualitative analysis for open ended questions) | □ | □ | □ | □ |
| **Results** |  |  |  |  |
| Were all relevant data reported? | □ | □ | □ | □ |
| Are quantitative results definitive (significant), and are relevant non-significant results also reported? | □ | □ | □ | □ |
| Have qualitative results been adequately interpreted (e.g. using an explicit theoretical framework), and have any quotes been properly justified and contextualised? | □ | □ | □ | □ |
| **Conclusions and discussion** |  |  |  |  |
| Have the researchers drawn an appropriate link between the data and their conclusions? | □ | □ | □ | □ |
| Have the findings been placed within the wider body of knowledge in the field (e.g. via a comprehensive literature review), and are any recommendations justified? | □ | □ | □ | □ |

From: (NICE 2012, -appendix E)


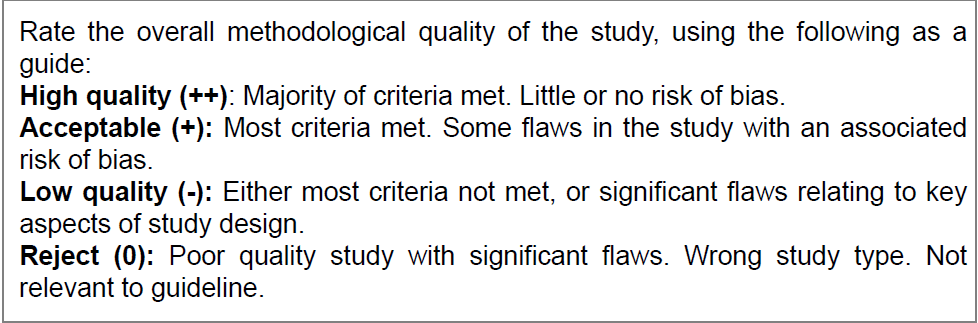


Rating scale from: (Roever 2015)

NICE. 2012. *Sickle Cell Acute Painful Episode: Management of an Acute Painful Sickle Cell Episode in Hospital*. Edited by National Institute for Health and Care Excellence: Guidelines. Vol. (NICE Clinical Guidelines, No. 143.) Appendix E, Evidence tables. Manchester (UK): National Institute for Health and Clinical Excellence (NICE)

Copyright © 2012, National Institute for Health and Clinical Excellence.

Roever, L. 2015. "Critical appraisal of a questionnaire study." *Evidence Based Medicine and Practice* 1 (2):1-2.
